# EXPLORING RELUCTANCE: HEALTH PROFESSIONS STUDENTS AND GERIATRIC CARE

**DOI:** 10.1093/geroni/igae098.3841

**Published:** 2024-12-31

**Authors:** Mingyang Zheng, Pamela Frasier, H George Philippi Jr.

**Affiliations:** Radford University, Blacksburg, Virginia, United States; Radford University, Radford, Virginia, United States; Radford University, Radford University, Virginia, United States

## Abstract

The aging population in the United States is growing rapidly, yet a shortage of healthcare workers persists, with health profession students showing limited interest in geriatric care. It is urgent to understand the reluctance of working with older adults among health profession students to develop interventions to address the workforce shortage issue. This study aims to understand the factors influencing these students’ intentions to work with older adults using the Theory of Planned Behavior. Using a cross-sectional survey design, the study surveyed 244 health profession students at a mid-sized public comprehensive university in the Southeast. Structural equation modeling demonstrated a good fit (χ² = 625.981, df = 340, p < 0.001, CFI = 0.918, RMSEA = 0.059, SRMR = 0.064, TLI = 0.908). The results indicated that attitudes (β = 0.930, p < 0.001) and subjective norms (β = 0.864, p < 0.001) play critical roles in shaping health profession students’ intention toward geriatric care, while perceived behavioral control (β = 0.169, p = 0.118) and self-efficacy (β = 0.008, p = 0.976) were not significant predictors. These findings suggest that educational interventions should focus on improving attitudes toward older adults and leveraging social influences to enhance the desirability of geriatric careers. Future research should explore longitudinal changes in health profession students’ attitudes and evaluate targeted interventions on students’ career intentions. Addressing biases and enhancing the educational curriculum are crucial steps toward mitigating the workforce shortage in geriatric care.

